# Supplementary material for: All-printed nanomembrane wireless bioelectronics using a biocompatible solderable graphene for multimodal human-machine interfaces
Source: Nat Commun. 2020 Jul 10;11:3450. doi: 10.1038/s41467-020-17288-0 (PMC7351733; doi:10.1038/s41467-020-17288-0)
Supplement: Supplementary file 1 — Supplementary Information [file 41467_2020_17288_MOESM1_ESM.pdf]

Supplementary Information

All-printed nanomembrane wireless bioelectronics using a biocompatible solderable graphene for multimodal human-machine interfaces

Young-Tae Kwon[1,6], Yun-Soung Kim[1,6], Shinjae Kwon[1], Musa Mahmood[1], Hyo-Ryoung Lim[1], Si-Woo Park[2], Sung-Oong Kang[2], Jeongmoon J. Choi[3], Robert Herbert[1], Young C. Jang[3,4], Yong-Ho Choa[2], and Woon-Hong Yeo[1,4,5]✉

[1]George W. Woodruff School of Mechanical Engineering, Institute for Electronics and Nanotechnology, Georgia Institute of Technology, Atlanta, GA 30332, USA.
[2]Department of Materials Science & Chemical Engineering, Hanyang University, Ansan 15588, South Korea.
[3]School of Biological Sciences, Georgia Institute of Technology, Atlanta, GA 30332, USA.
[4]Wallace H. Coulter Department of Biomedical Engineering, Parker H. Petit Institute for Bioengineering and Biosciences, Georgia Institute of Technology, Atlanta, GA 30332, USA.
[5]Neural Engineering Center, Flexible and Wearable Electronics Advanced Research, Institute for Materials, Institute for Robotics and Intelligent Machines, Georgia Institute of Technology, Atlanta, GA 30332, USA.
[6]These authors contributed equally: Young-Tae Kwon, Yun-Soung Kim.
✉e-mail: whyeo@gatech.edu

The PDF file includes:

Supplementary Figure 1. AJP procedure for the multi-layered, wearable electronics.
Supplementary Figure 2. Sequential cross-sectional profiles of the electrodes printed via AJP.
Supplementary Figure 3. Sequential cross-sectional profiles of the circuit printed via AJP.
Supplementary Figure 4. Stiffness of the elastomer.
Supplementary Figure 5. Printed circuit design.
Supplementary Figure 6. Description of battery connection.
Supplementary Figure 7. Characterization of the FCR dispersed in aqueous ink.
Supplementary Figure 8. EMG characterization depending on the size of printed FCR electrodes.
Supplementary Figure 9. Structural characterizations for the printed Ag and Au electrodes.
Supplementary Figure 10. Mechanical bending characterization of the printed FCR electrodes.
Supplementary Figure 11. Mechanical stretching characterization of the printed FCR electrodes.
Supplementary Figure 12. Biocompatible characterizations for the printed electrodes.
Supplementary Figure 13. Sequential SEM images of the photonic-sintered Ag layer.
Supplementary Figure 14. Elemental composition comparison for the sintered Ag membrane.
Supplementary Figure 15. Mechanical bending characterization of the fully printed circuit.
Supplementary Figure 16. Device functionality for the integrated EMG system.
Supplementary Figure 17. Mapping process to generate heat-maps.
Supplementary Figure 18. Overview of wearable flexible printed electronics for machine interfaces.
Supplementary Figure 19. Flowchart for a machine interface, enabled by single- and multi-channel device.
Supplementary Figure 20. Acceleration signals from an arm rotation in z axis.
Supplementary Figure 21. Real-time confusion matrix for drone and RC car.
Supplementary Figure 22. Real-time confusion matrix for PowerPoint navigator.
Supplementary Figure 23. EMG signals from multi-channel, printed system.
Supplementary Table 1. Ink material, printing, curing/sintering parameters.
Supplementary Table 2. List of chip components used in fully printed circuit.
Supplementary Table 3. Characterization of impedance and SNR for four different electrodes.
Supplementary Table 4. Summary for the reliability of p-NHE.
Supplementary Table 5. Elemental composition of the sintered Ag membrane without and with the FCR layer.
Supplementary Table 6. RSSI response of commercial rigid and printed circuits.
Supplementary Table 7. The fabrication yield at each process.
Supplementary Table 8. Classification for CNN.
Supplementary Table 9. List of commands for controlling the drone, RC car, and PowerPoint navigator.
Supplementary Table 10. Comparison of average RMS values from synchronized multi-device EMG recording.

Supplementary Figures

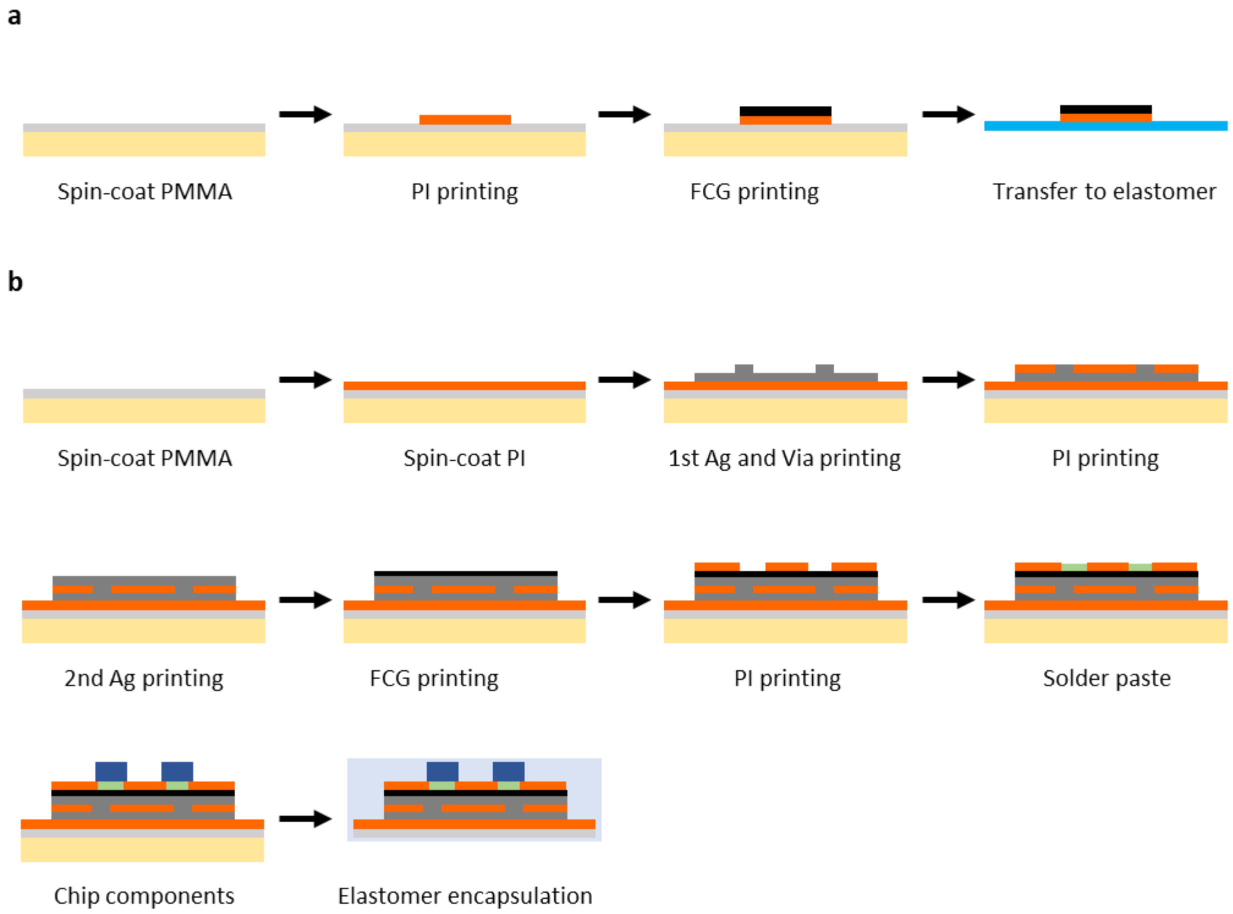

Supplementary Figure 1. AJP procedure for the multi-layered, wearable electronics. a, Fabrication of the electrodes with the PI and FCR materials on the elastomer. **b**, Printing process for the multi-layered circuit with the multi-materials, including the PI, Ag, and FCR.

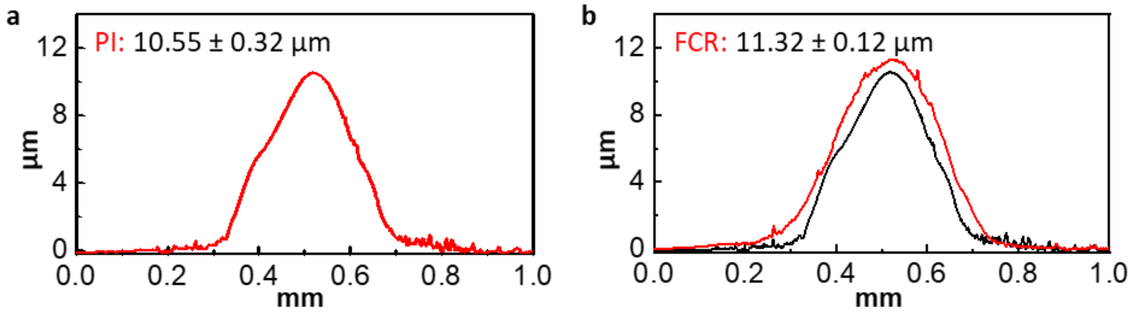

Supplementary Figure 2. Sequential cross-sectional profiles of the electrodes printed via AJP. a, In Step 1, a 10.55 µm-thick PI support layer is printed. **b**, In Step 2, a 0.77 µm-thick FCR conductive layer is printed.

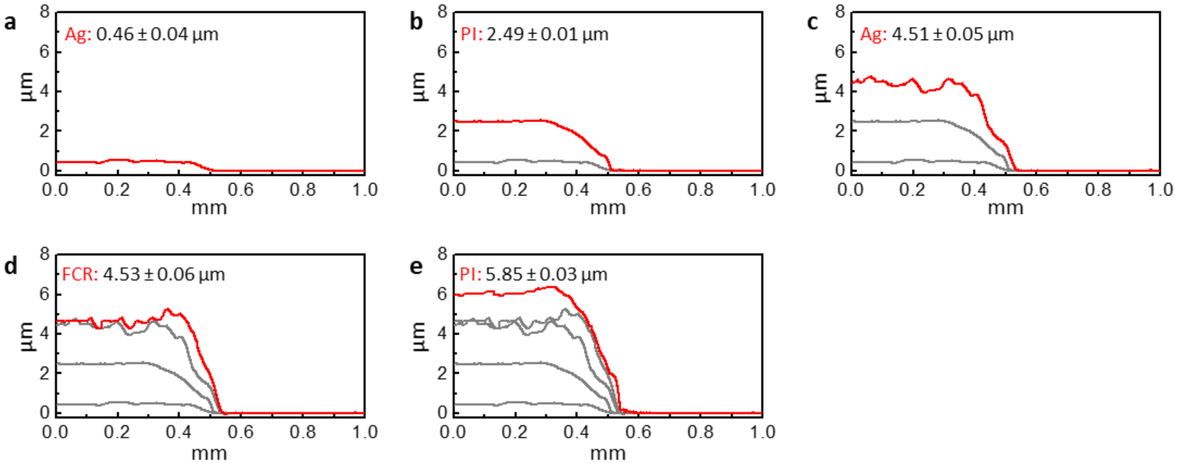

Supplementary Figure 3. Sequential cross-sectional profiles of the circuit printed via AJP. a, In Step 1, a 0.46 µm-thick Ag conductive layer is printed. **b**, In Step 2, a 2.03 µm-thick PI dielectric layer is printed. **c**, In Step 3, a 2.02 µm-thick Ag conductive layer is printed. **d**, In Step 4, a 0.1 µm-thick FCR layer is printed. **e**, In Step 5, a 1.32 µm-thick PI dielectric is printed.

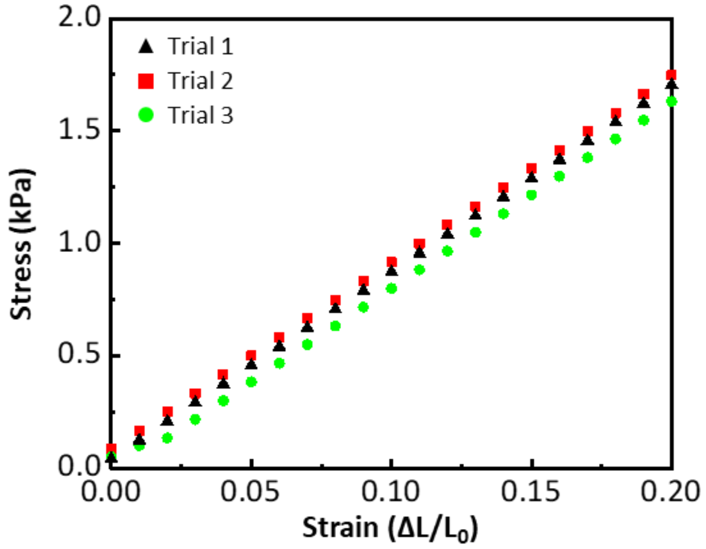

Supplementary Figure 4. Stiffness of the elastomer. Stress vs. strain curves of the Ecoflex from 3 trials. Young's modulus is determined by the slope of each curve in its linear regime. The average modulus is 8.47 kPa (1st trial: 8.74 kPa, 2nd trial: 8.16 kPa, 3rd trial: 8.52 kPa).

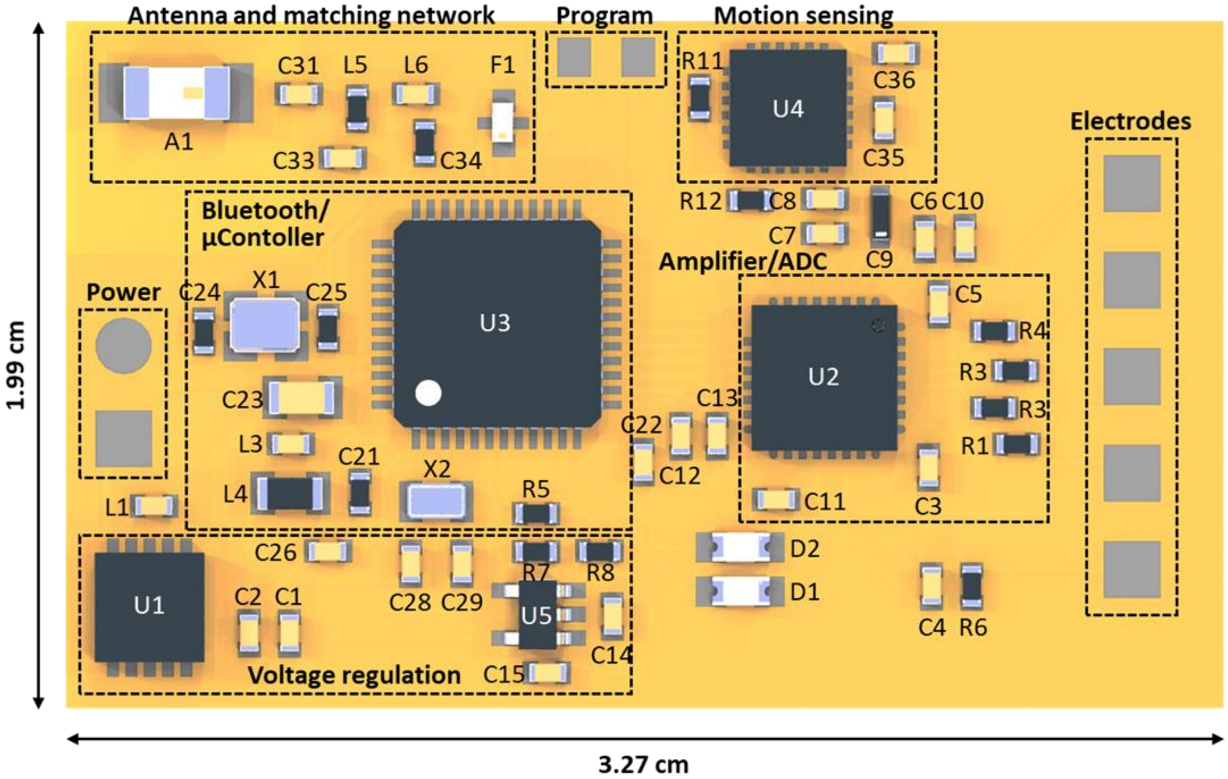

Supplementary Figure 5. Printed circuit design. Top-view illustration of fully printed circuit component with highlighted functional blocks. Detail list of the chip components is shown in Supplementary Table 1.

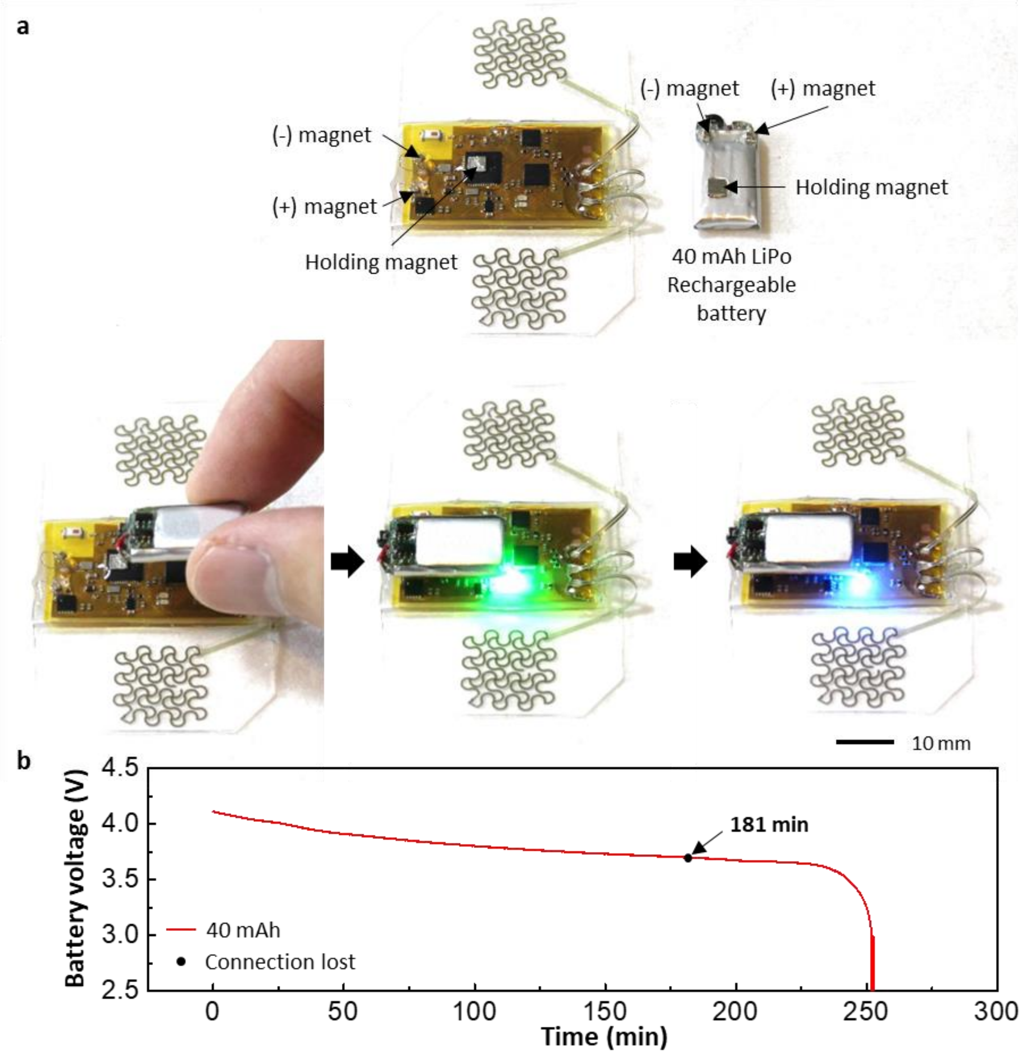

Supplementary Figure 6. Description of battery connection. a, Photographs showing the LiPo battery connection with neodymium magnets. **b,** Battery life test for 40 mAh capacity.

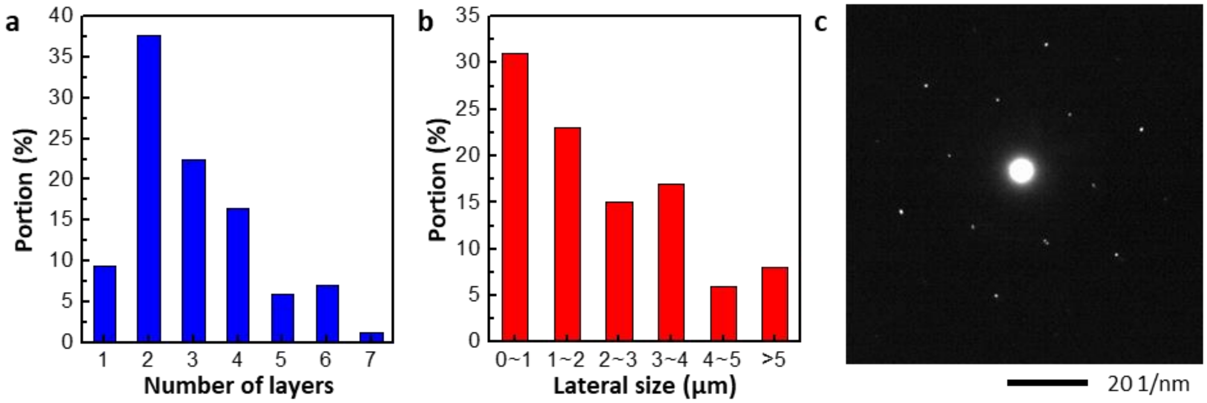

Supplementary Figure 7. Characterization of the FCR dispersed in aqueous ink. a,b, Number of layers (**a**) and lateral size (**b**) of FCR materials. 100 FCR sheets are counted from AFM results. **c**, Selected area ED pattern at the basal plane of FCR.

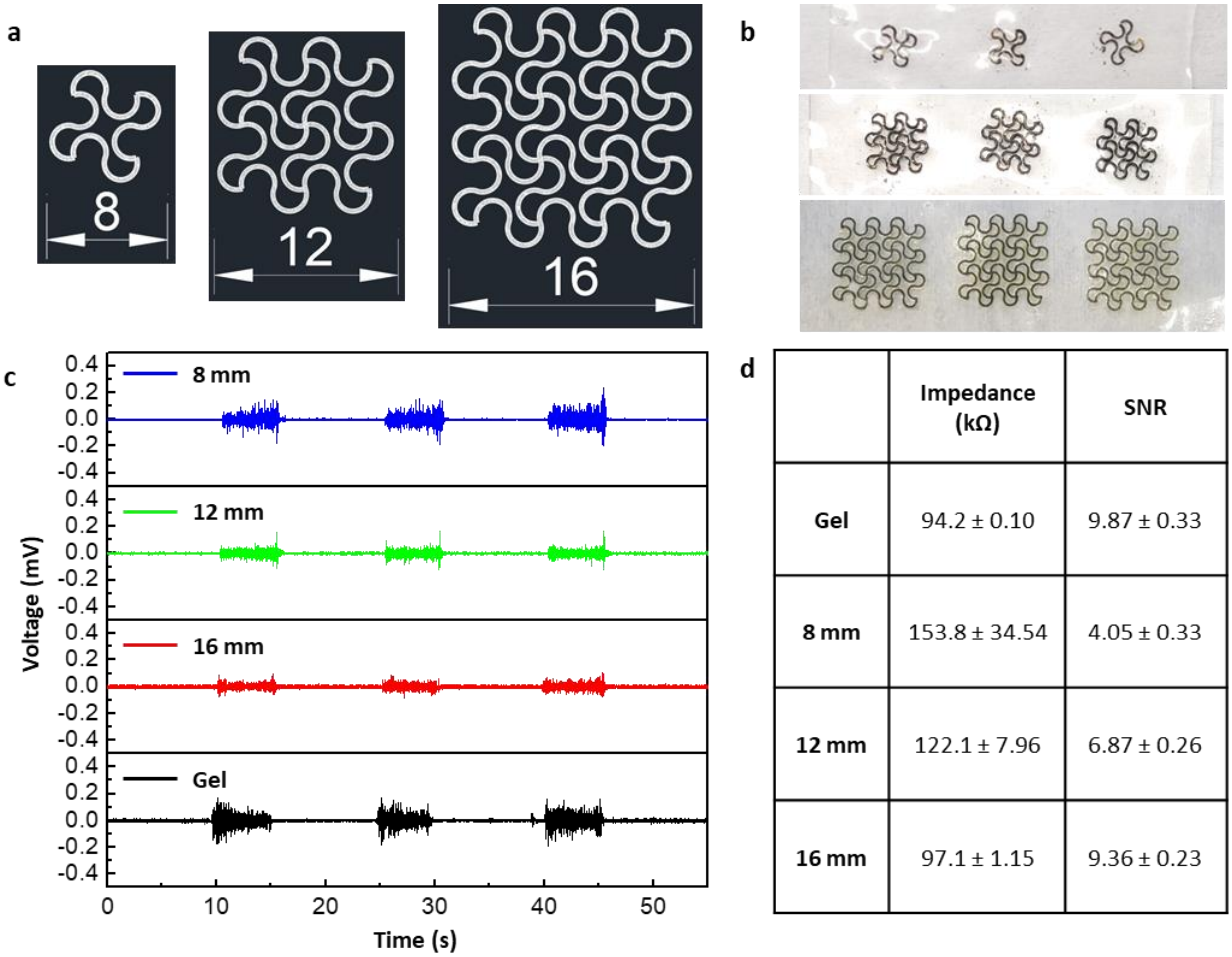

	Impedance (kΩ)	SNR
Gel	94.2 ± 0.10	9.87 ± 0.33
8 mm	153.8 ± 34.54	4.05 ± 0.33
12 mm	122.1 ± 7.96	6.87 ± 0.26
16 mm	97.1 ± 1.15	9.36 ± 0.23

Supplementary Figure 8. EMG characterization depending on the size of printed FCR electrodes. a, Electrode design to optimize the size of printed FCR ranging from 8 to 16 mm. **b**, Photos showing the FCR electrodes printed with **a**. **c**, EMG characteristics measured from three different sized FCR electrodes and conventional gel. **d**, Summary of the measured impedance and SNRs for three different sized FCR electrodes and conventional gel.

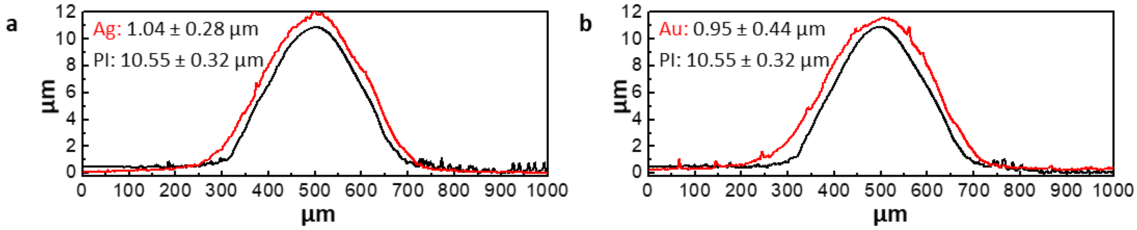

Supplementary Figure 9. Structural characterizations for the printed Ag and Au electrodes. **a,b**, Cross-sectional profiles for the printed Ag/PI (**a**) and Au/PI (**b**) electrodes.

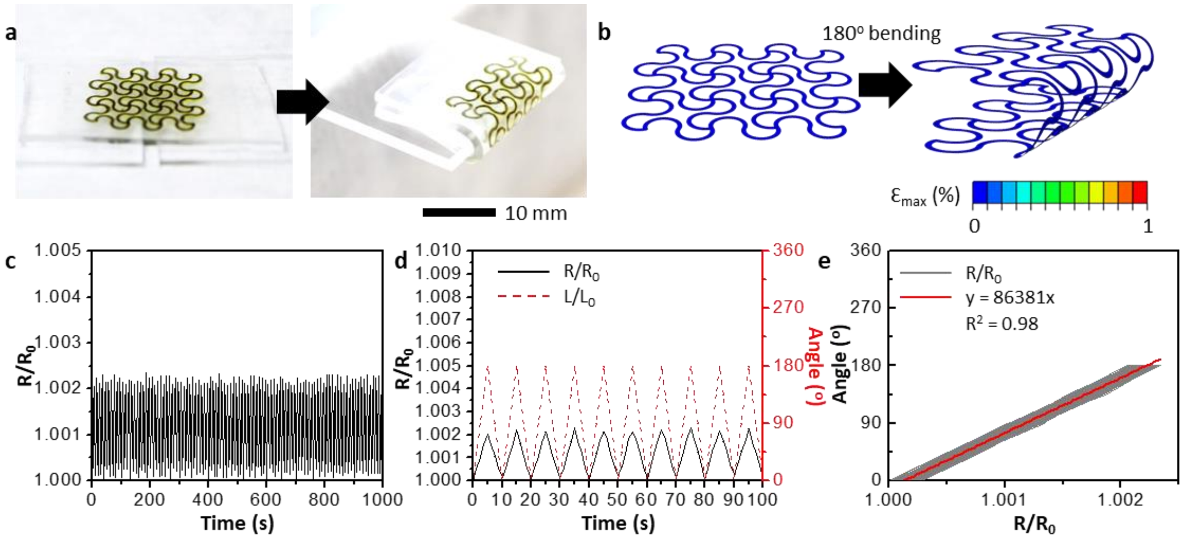

Supplementary Figure 10. Mechanical bending characterization of the printed FCR electrodes. a, Photos showing mechanical flexibility of the electrodes (radius of curvature: 1.5 mm). **b**, FEA results of printed electrodes before (left) and after (right) 180° bending. **c**, Relative resistance change of an electrode upon cyclic 180° bending for 100 times. **d**, Graph representing relative resistance change (black) according to bending change (red) for 10 cycles. **e**, Correlation of data obtained from relative resistance vs. applied bending.

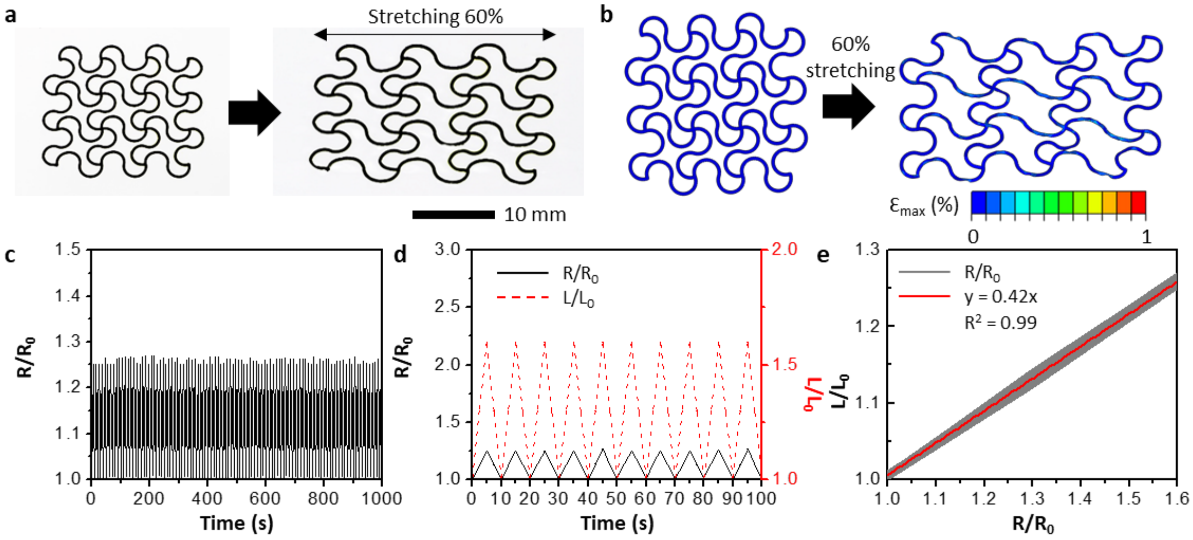

Supplementary Figure 11. Mechanical stretching characterization of the printed FCR electrodes. a, Photos showing mechanical stretchability of the electrodes. **b**, FEA results of printed electrodes before (left) and after (right) 60% stretching **c**, Relative resistance change of an electrode upon cyclic 60% stretching for 100 times. **d**, Graph representing relative resistance change (black) according to strain change (red) for 10 cycles. **e**, Correlation of data obtained from relative resistance vs. applied strain.

a

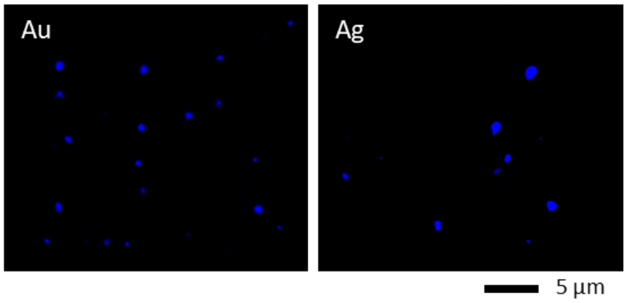

b

Membranes	Absorbance (OD)	Fluorescence (OD)
Control	1.54 ± 0.05	573.7 ± 4.1
Elastomer	1.50 ± 0.03	570.6 ± 5.5
FCR	1.52 ± 0.03	572.1 ± 3.2
Au	1.49 ± 0.02	571.4 ± 2.3
Ag	1.22 ± 0.05	503.0 ± 2.6

Supplementary Figure 12. Biocompatible characterizations for the printed electrodes. a, Optical microscopic images of keratinocyte cells after exposure to original DMEM (control) and DMEM extracts of the Au and Ag for 7 days. **b**, Summary of the measured cell absorbance and fluorescence for five different membranes, including the control, elastomer, printed FCR, Au, and Ag materials.

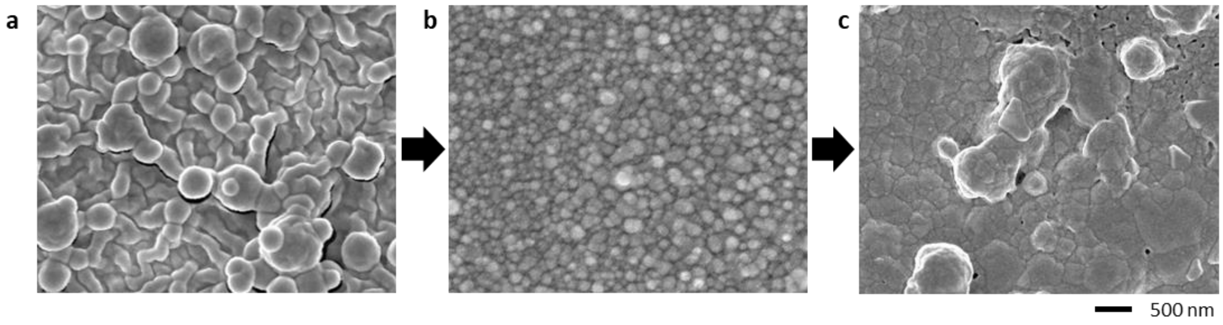

Supplementary Figure 13. Sequential SEM images of the photonic-sintered Ag layer. a-c, Micro-structure of as-printed (**a**), sintered with 2 kV/2 ms (**b**), and multiple-sintered with 2 kV/2 ms/5 times (**c**). **c** is optimal photonic sintering condition for the printed Ag membrane.

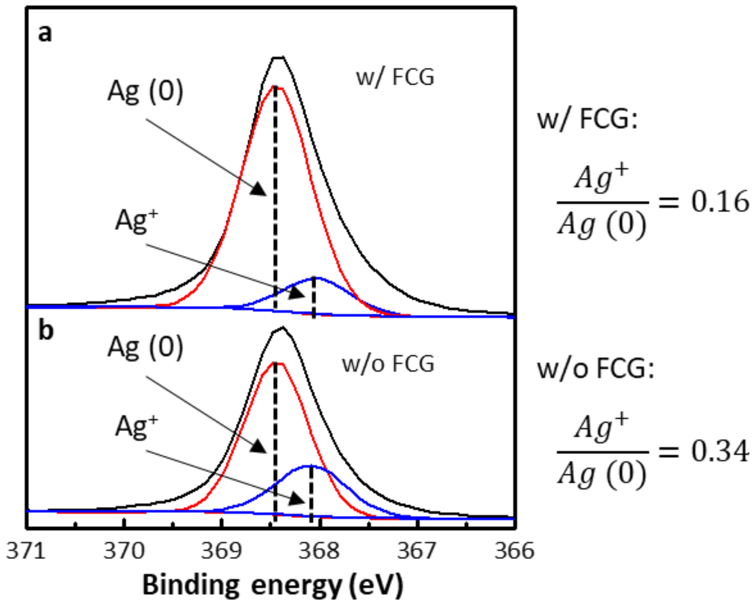

Supplementary Figure 14. Elemental composition comparison for the sintered Ag membrane. a,b, XPS data of the sintered Ag membrane with (**a**) and without (**b**) the FCR layer. High-resolution XPS spectra peaks are curve-fitted with Ag metal (368.4 eV) and Ag ion (368.1 eV).

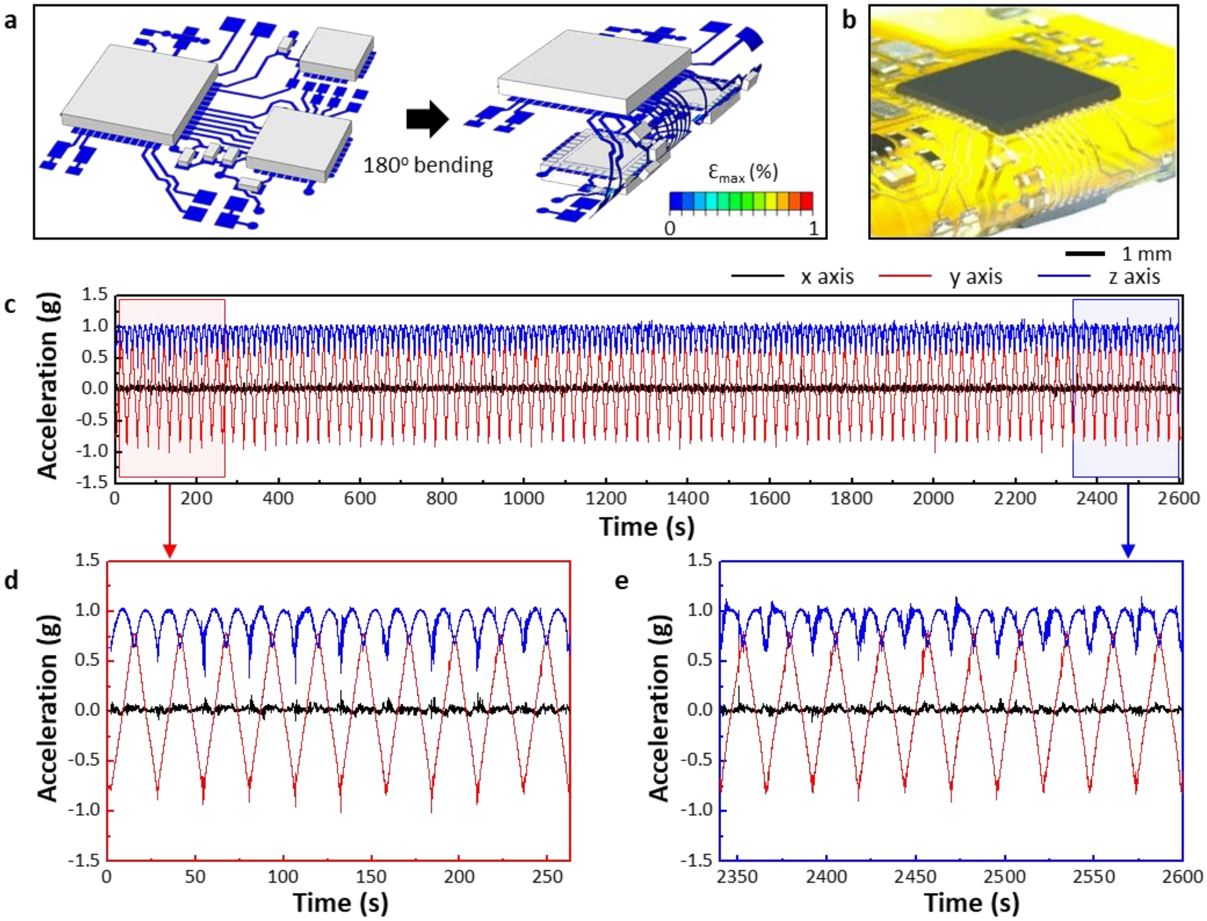

Supplementary Figure 15. Mechanical bending characterization of the fully printed circuit. a, FEA results of printed circuit before (left) and after 180° bending. **b**, Optical image showing the mechanical flexibility of the printed circuit (radius of curvature: 1.5 mm). **c**, Three-axis acceleration data obtained during 100 cyclic bending, showing that the device could maintain its power and connectivity. **d,e**, Acceleration data of first (**d**) and last (**e**) ten bending cycles.

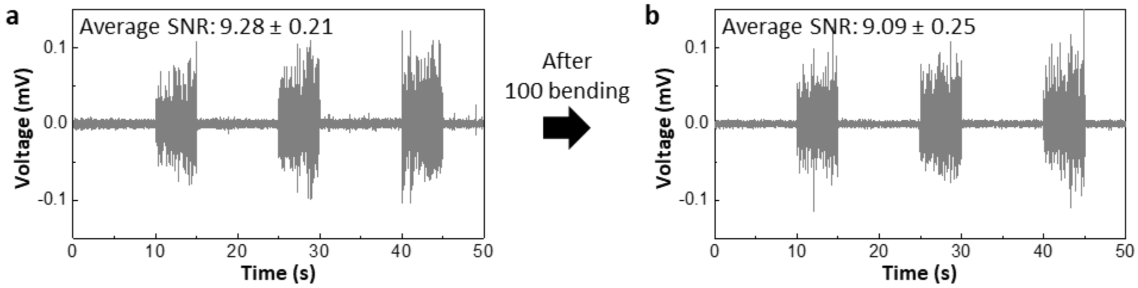

Supplementary Figure 16. Device functionality for the integrated EMG system. a,b, Forearm EMG characteristics of the integrated system before (**a**) and after (**b**) 100 bending.

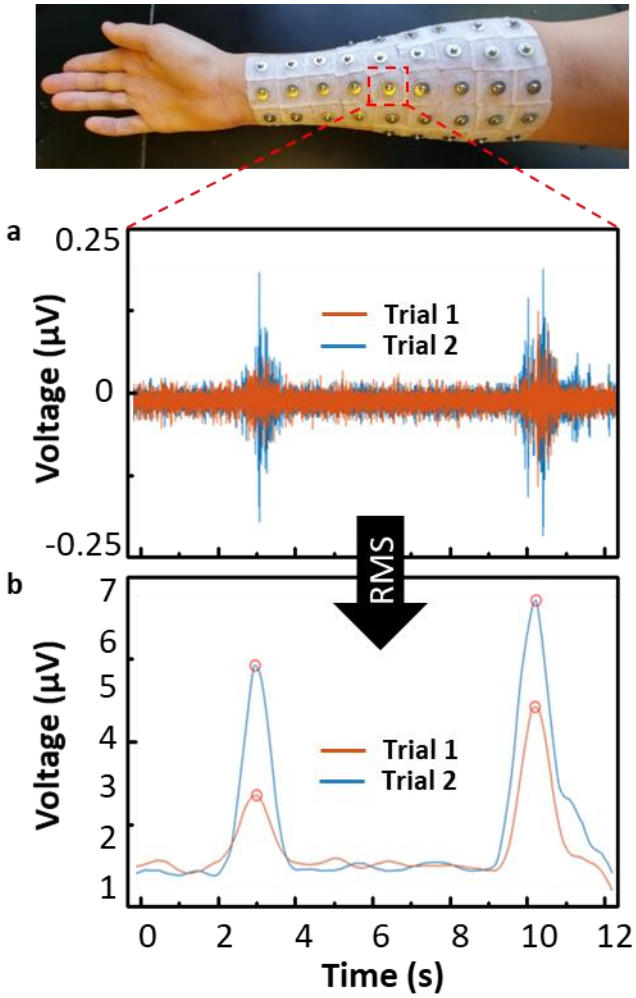

Supplementary Figure 17. Mapping process to generate heat-maps. Top photo shows the mapping method for analyzing the muscles on the forearm. **a,b**, Representative EMG signals (**a**) and RMS values (**b**) with 2 trials, measured from one of the electrodes

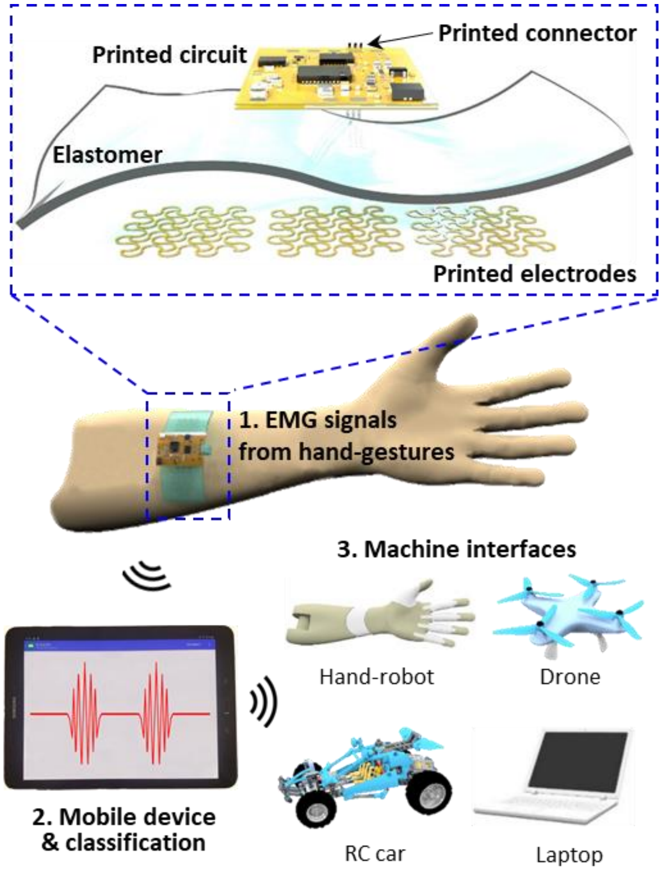

Supplementary Figure 18. Overview of wearable flexible printed electronics for machine interfaces. Illustrations show the principles of the HMIs employed in this study. The EMG signals generated from hand-gestures are wirelessly transmitted to the mobile device (1). The acquired data is classified via smart signal processing algorism (2) and wirelessly transmitted to the target machines, including the robotic hand, drone, RC car, and Laptop. Enlarged sketch captures the main structural components and their assembly for the fully printed electronics.

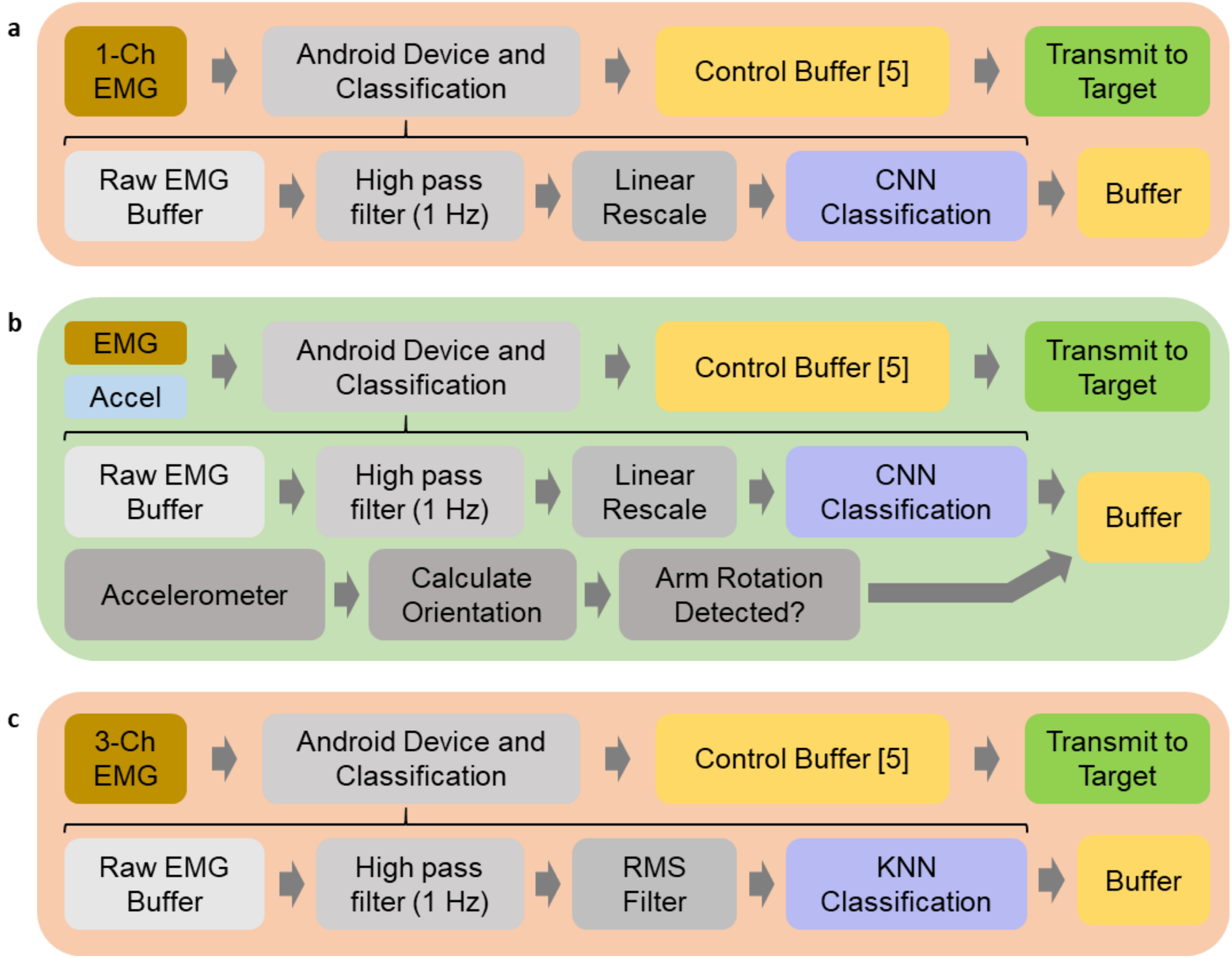

Supplementary Figure 19. Flowchart for a machine interface, enabled by single- and multi-channel device. a-c, EMG HMI procedures for PowerPoint (**a**), RC car and Drone (**b**), and robotic hand (**c**).

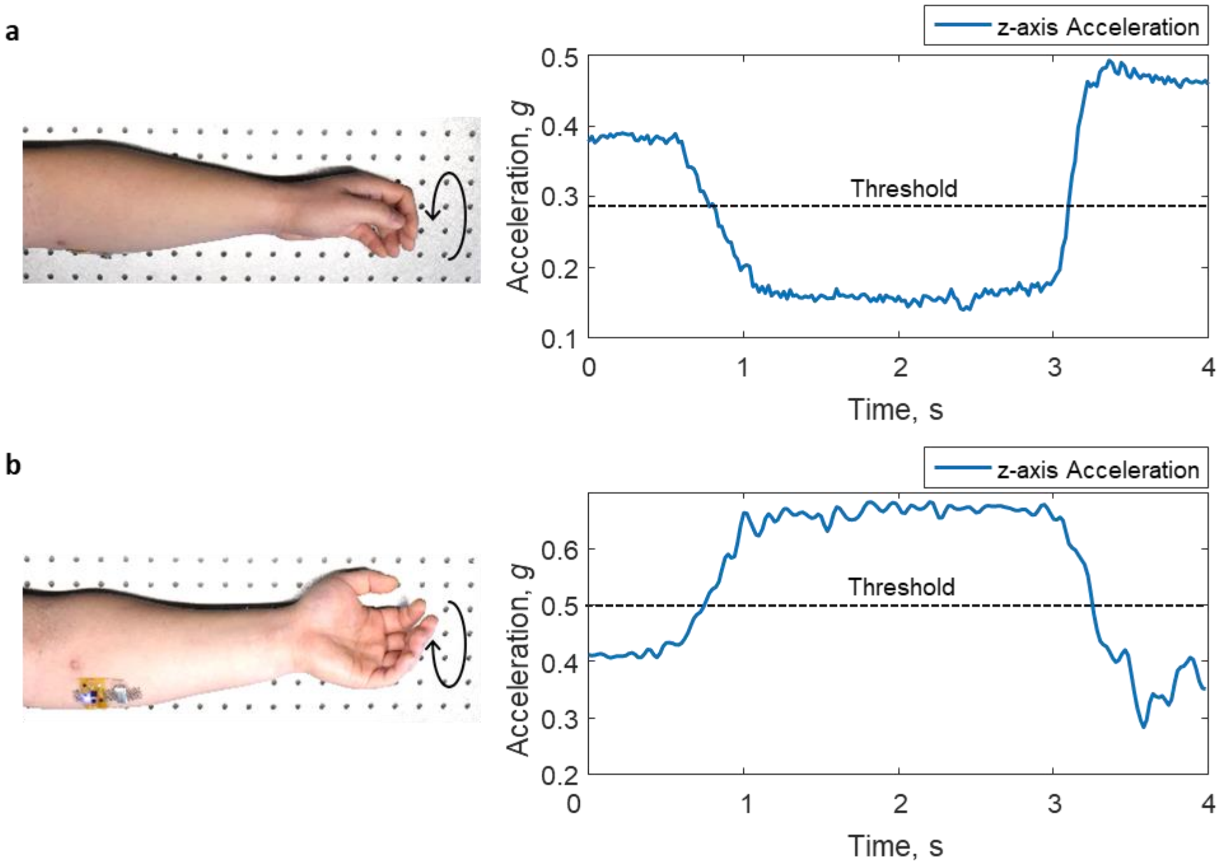

Supplementary Figure 20. Acceleration signals from an arm rotation in z axis. a,b, The two gestures, including rotate arm clockwise (**a**)/counterclockwise (**b**), used to control the drone and RC car, along with the corresponding acceleration signals in z axis.

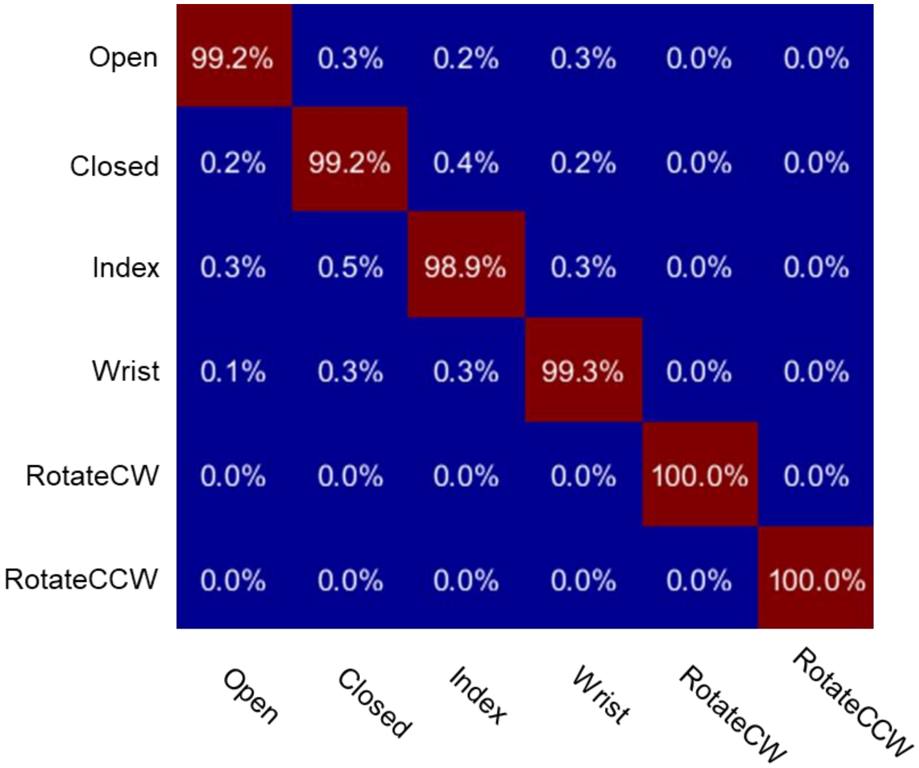

Supplementary Figure 21. Real-time confusion matrix for drone and RC car. Classification results from 10 trials shows high accuracy of 99.43% across all classes.

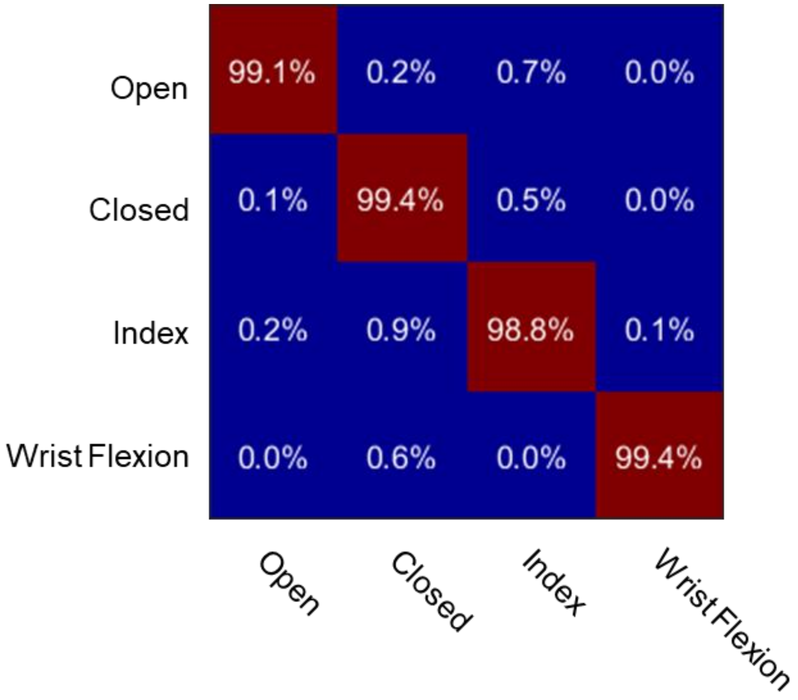

Supplementary Figure 22. Real-time confusion matrix for PowerPoint navigator. Classification results from 10 trials shows high accuracy of 99.17% across all classes.

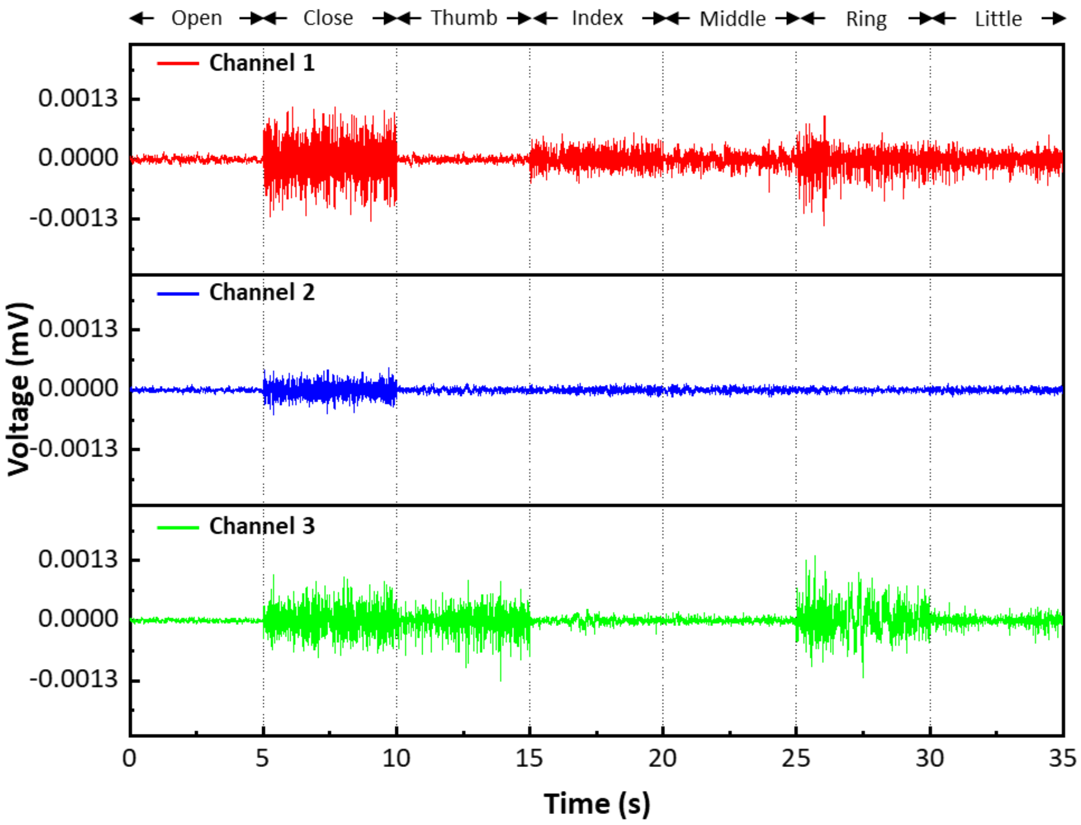

Supplementary Figure 23. EMG signals from multi-channel, printed system. Multi-channel EMG values measured from 7 different hand gestures, including hand open/closed, thumb, index, middle, ring, and little, shown in Figure 4e.

Supplementary Tables

Supplementary Table 1. Ink material, printing, curing/sintering parameters. The table listed the detail conditions, including the solvent, viscosity, sheath/atomization rate, nozzle diameter, AJP speed, stage temperature during printing, and curing/sintering condition.

**Parameter**	**PI**	**FCR**	**Ag**
Solvent	N-Methyl-2-Pyrrolidone	Ethanol	DI water
Viscosity (cP)	350	5.4	15
Sheath rate (ccm)	20	30	30
Atomization rate (ccm)	1000 (exhaust)<br>1100 (atomization)	40	25
Nozzle diameter (μm)	300	200	200
Printing speed (mm/s)	10	10	10
Stage temperature (^{o}C)	80	70	70
Curing or sintering condition	Thermal curing<br>at 250 ^{o}C for 1 h	Thermal curing<br>at 100 ^{o}C for 1 h	Photonic sintering<br>at 2 kV, 2 ms, and 5 times

Supplementary Table 2. List of chip components used in fully printed circuit. The table describes the components, description, value, and part number Multi-channel EMG values.

Component	Description	Value	Part number
U1	3.3 voltage regulator	N/A	TPS63001
U2	Analog front end	N/A	ADS1292
U3	Bluetooth PSoC	N/A	NPF52832-QFAA-R
U4	Motion sensor	N/A	MPU22941
U5	Current limit active low load switch	N/A	N/A
L1	0402 inductor	2.2 μH	N/A
L3	0402 inductor	15 nH	N/A
L4	0603 inductor	10 μH	N/A
L5	0402 inductor	10 nH	N/A
L6	0402 inductor	2.7 nH	N/A
C1, C10, C14, C15	0402 ceramic capacitor	10 μF	N/A
C2	0402 ceramic capacitor	22 μF	N/A
C3,C5	0402 ceramic capacitor	4.7 nF	N/A
C4	0402 ceramic capacitor	1.0 nF	N/A
C6, C7, C12, C22, C26, C33, C35, C36	0402 ceramic capacitor	0.1 μF	N/A
C8, C11, C13	0402 ceramic capacitor	1.0 μF	N/A
C9	0402 tantalum capacitor	1.0 μF	N/A
C21	0402 ceramic capacitor	4.7 μF	N/A
C23	0603 ceramic capacitor	10 μF	N/A
C24, C25, C28, C29	0402 ceramic capacitor	12 pF	N/A
C31	0402 ceramic capacitor	0.4 pF	N/A
C34	0402 ceramic capacitor	100 pF	N/A
R1, R2, R3, R4	0402 resistor	30 kΩ	N/A
R5, R6, R7, R8	0402 resistor	1 MΩ	N/A
R11, R12	0402 resistor	10 kΩ	N/A
A1	2.45 GHz RF chip antenna	N/A	2450AT18A100
F1	2.45 GHz low pass filter	N/A	S450FM07A0029
X1	32 MHz crystal	N/A	ECS-320-8-37CKM
X2	32.768 kHz crystal	N/A	ECS-.327-9-12-TR

Supplementary Table 3. Characterization of impedance and SNR for four different electrodes. Four electrodes include conventional gel, printed Ag, Au, and FCR electrodes. The SNR summarized the detail value shown in Fig. 2k.

	Impedance (kΩ)	SNR
**Gel**	93.1 ± 0.57	9.87 ± 0.33
**Ag**	101.2 ± 1.32	8.40 ± 0.34
**Au**	103.3 ± 1.10	8.94 ± 0.09
**FCR**	97.8 ± 1.03	9.36 ± 0.23

Supplementary Table 4. Summary for the reliability of p-NHE. a, Structural reliability of the electrode and the circuit calculated from Fig. 3g and Supplementary Figs 10, 11. Functional reliability of the integrated system calculated from Supplementary Figs 15, 16.

a

	**Electrodes**		**Circuit**
	**Stretching** (% change after 100 cycles)	**Bending** (% change after 100 cycles)	**Bending** (% change after 100 cycles)
R/R_0	0.1	0.2	0.1

b

	**Integrated system**
	**Bending** (% change after 100 cycles)
Acceleration	x: 0.3, y: 1.2, z: 1.1
EMG SNR	2.0

Supplementary Table 5. Elemental composition of the sintered Ag membrane without and with the FCR layer. The elemental percentage is obtained from XPS results.

	w/o FCR (atomic %)	w/ FCR (atomic %)
**Ag3d**	70.6	83.3
**O1s**	18.3	4.2
**C1s**	11.1	12.5

Supplementary Table 6. RSSI response of commercial rigid and printed circuits. The table describes the RSSI values according to the distance between circuit and receiver.

Distance (m)	Rigid circuit (dBm)	Printed circuit (dBm)
0	-41.2 ± 1.7	-40.4 ± 2.1
1	-56.5 ± 3.1	-55.6 ± 5.3
2	-71.0 ± 5.1	-73.2 ± 4.2
3	-75.2 ± 3.9	-77.4 ± 2.7
4	-78.9 ± 5.4	-81.8 ± 6.3
5	-80.2 ± 4.4	-81.6 ± 3.5
6	-84.6 ± 3.9	-83.2 ± 3.9
7	-85.5 ± 2.0	-84.0 ± 4.0
8	-85.6 ± 4.0	-88.6 ± 4.4
9	-88.3 ± 3.9	-88.0 ± 3.7
10	-86.4 ± 3.2	-88.2 ± 4.4
11	-84.9 ± 2.7	-89.4 ± 4.6
12	-88.7 ± 4.2	-88.8 ± 1.6
13	-90.5 ± 3.2	-89.6 ± 3.8
14	-90.1 ± 6.4	-91.0 ± 2.5
15	-93.5 ± 7.2	-90.4 ± 2.9

Supplementary Table 7. The fabrication yield at each process. The table indicates the yields for printing, soldering, and integration process with the optimized conditions.

	**Printing**		**Soldering**	**Integration**
	**Sensor**	**Circuit**		
**Yield (%)<br>(Operations/Trials)**	93<br>(9.3/10)	90<br>(9/10)	100<br>(9/9)	67<br>(6/9)

Supplementary Table 8. Classification for CNN. 2-layer CNN for classifying 0.512 second data from 1-channel EMG.

Layer Name	Layer Description
Input	Time Domain Input: [length(w) x 1]
ConV1 (1D)	[8] conv, stride [1], 32 LeakyReLU Units (α = 0.1)
ConV2 (1D)	[4] conv, stride [2], 64 LeakyReLU Units (α = 0.2)
FC	Fully Connected Layer, 256 ReLU Units, 50% Dropout
Softmax	4-way softmax output

Supplementary Table 9. List of commands for controlling the drone, RC car, and PowerPoint navigator. All machine interface could be controlled by the EMG (Open, closed, index, and wrist flexion) and acceleration (Rotate CW/CCW) signals.

Machine / Gesture	**Drone**	**RC-car**	**PPT**
**Open**	Stop	Stop	N/A
**Closed**	Forward	Forward	Next slide
**Index**	Rotate right	Backward	Previous slide
**Wrist flexion**	Take off & land	N/A	Start & stop PPT
**Rotate CW**	N/A	Rotate right	N/A
**Rotate CCW**	N/A	Rotate left	N/A

Supplementary Table 10. Comparison of average RMS values from synchronized multi-device EMG recording. The table lists the RMSs calculated from Supplementary Fig. 23.

	Channel 1 (x10^{-4} V)	Channel 2 (x10^{-4} V)	Channel 3 (x10^{-4} V)
**Close**	0.48	0.31	0.29
**Thumb**	4.00	1.42	2.72
**Index**	0.44	0.45	2.19
**Middle**	1.65	0.48	0.70
**Ring**	1.22	0.46	0.42
**Little**	1.92	0.35	3.24
