# Supplementary material for: All-printed nanomembrane wireless bioelectronics using a biocompatible solderable graphene for multimodal human-machine interfaces
Source: Nat Commun. 2020 Jul 10;11:3450. doi: 10.1038/s41467-020-17288-0 (PMC7351733; doi:10.1038/s41467-020-17288-0)
Supplement: Supplementary file 6 — Description of Additional Supplementary Files [file 41467_2020_17288_MOESM6_ESM.docx]

Description of Additional Supplementary Files

File: Supplementary Movie 1

Description: Human-drone interface using a single-channel device.

File: Supplementary Movie 2

Description: Human-RC car interface using a single-channel device.

File: Supplementary Movie 3

Description: Human-PPT interface using a single-channel device.

File: Supplementary Movie 4

Description: Human-hand robot interface using multiple channel devices.
